# Error in Results, Discussion, and Table

**DOI:** 10.1001/jamanetworkopen.2021.32776

**Published:** 2021-10-01

In the Research Letter titled “Validation of an At-Home Direct Antigen Rapid Test for COVID-19,”^1^ published August 27, 2021, the combined sensitivity of the direct antigen rapid test should have been 96.2% (95% CI, 88.8%-100.0%), not 96.3% (95% CI, 89.5%-100.0%). These numbers appeared in the Results, Discussion, and Table. This article has been corrected.^1^
